# Discovery-2: an interactive resource for the rational selection and comparison of putative drug target proteins in malaria

**DOI:** 10.1186/1475-2875-12-116

**Published:** 2013-03-28

**Authors:** Phelelani T Mpangase, Michal J Szolkiewicz, Misha le Grange, Jeanré H Smit, Pieter B Burger, Fourie Joubert

**Affiliations:** 1 Bioinformatics and Computational Biology Unit, Department of Biochemistry, University of Pretoria, Pretoria 0001, South Africa

## Abstract

**Background:**

Drug resistance to anti-malarial compounds remains a serious problem, with resistance to newer pharmaceuticals developing at an alarming rate. The development of new anti-malarials remains a priority, and the rational selection of putative targets is a key element of this process. Discovery-2 is an update of the original Discovery *in silico* resource for the rational selection of putative drug target proteins, enabling researchers to obtain information for a protein which may be useful for the selection of putative drug targets, and to perform advanced filtering of proteins encoded by the malaria genome based on a series of molecular properties.

**Methods:**

An updated *in silico* resource has been developed where researchers are able to mine information on malaria proteins and predicted ligands, as well as perform comparisons to the human and mosquito host characteristics. Protein properties used include: domains, motifs, EC numbers, GO terms, orthologs, protein-protein interactions, protein-ligand interactions. Newly added features include drugability measures from ChEMBL, automated literature relations and links to clinical trial information. Searching by chemical structure is also available.

**Results:**

The updated functionality of the Discovery-2 resource is presented, together with a detailed case study of the *Plasmodium falciparum* S-adenosyl-L-homocysteine hydrolase (PfSAHH) protein. A short example of a chemical search with pyrimethamine is also illustrated.

**Conclusion:**

The updated Discovery-2 resource allows researchers to obtain detailed properties of proteins from the malaria genome, which may be of interest in the target selection process, and to perform advanced filtering and selection of proteins based on a relevant range of molecular characteristics.

## Background

Drug resistance to current anti-parasitic compounds has become widespread and is on the increase, including resistance to newer treatments such as artemisinin [1,2]. While in-depth studies are ongoing on a relatively small number of selected putative targets for future exploitation, not many resources are available that focus on performing data mining and target identification on the complete malaria genome, in concert with relations to chemical compounds. Currently available resources that may be useful for target identification include PlasmoDB [3], TDR Targets [4], PlasmoMap [5], the Tropical Diseases Kernel [6] and also the original version of Discovery [7].

Recent approaches have illustrated the value of predicting the association of chemical compounds with putative protein drug targets, especially when the targets of compounds such as the GSK dataset with known activity against the parasite may be extrapolated using protein-ligand interaction databases such as ChemProt [8,9]. The Discovery resource attempts to use a similar approach in associating chemical compounds with malaria proteins using sequence homology, and also selective chemical similarity searches. While the resource is focused primarily at *Plasmodium falciparum*, it contains information for all proteins from *Plasmodium vivax*, *Plasmodium yoelii*, *Plasmodium knowlesi*, *Plasmodium chabaudi* and *Plasmodium berghei* and also for the human and mosquito hosts. Protein information includes sequences and annotations from PlasmoDB, Ensembl and VectorBase, functional predictions, gene ontology terms, orthology information, structural information, metabolic pathways, predicted putative protein-ligand interactions, druggability predictions, and literature links. The resource also contains chemical compounds from the ChEMBL database with chemical search functionality and putative ligand-protein prediction information. Protein searches may be performed using accession numbers, keywords or an advanced multi-parameter filtering interface. Chemical searches may be performed using keywords, SMILES strings or chemical structures.

This new implementation of Discovery (version 2) is a complete rewrite of the original Python-based system using Java and NetBeans with the implementation of automated updates based largely on web services. The previous version included information related to sequence features, orthology, ontology terms, structural information, metabolic pathways and protein ligand interactions. The new functionalities are the addition of expression information, literature information from PubMed abstracts, the implementation of druggability predictions from DrugEBIlity where available, the inclusion of malaria-related data from the clinical trials database and the implementation of Wiki-like user annotations. Specific advantages of Discovery-2 include extensive functionality to identify putative associations of proteins with ligands, an advanced chemical structure search interface encompassing the content of the ChEMBL database and an interactive system for refining putative target selections based on data mining of molecular properties. Additionally, the resource also contains data for the mosquito and human hosts, for easy comparative analysis.

## Methods

Discovery-2 was developed in Java with NetBeans. The protein sequences for *P. berghei*, *P. chabaudi*, *P. falciparum*, *P. knowlesi*, *P. vivax* and *P. yoelii* were downloaded from PlasmoDB (currently version 8.1). The *Homo sapiens* and *Anopheles gambiae* proteins were downloaded from Ensembl (currently version 64) [10] and VectorBase (currently version 3.7) [11], respectively. UniProt accessions were assigned to the proteins using an identifier mapping file downloaded from UniProt [12]. The UniProt accessions were used to assign Gene Ontology (GO) annotations from UniProt-GOA database (currently October 2012) [13]. Each GO term was linked to the Gene Ontology database [14] and the AmiGO visualization tool [15] is used to view the GO terms [16]. To detect orthologous genes between the different species, ortholog clusters were generated with OrthoMCL using mostly default parameters with a percentage match cut-off of 50% [17]. Protein families, domains and functional sites were identified using InterProScan, which uses an ensemble of different methods to automatically annotate the function and structure of protein sequences [18]. To identify possible structures of related proteins, a sequence similarity search against the PDB database was carried out using NCBI BLAST, with an E-value cut-off value of 10^-6^. Predicted 3D protein structures for *P. falciparum* and *Homo sapiens* were downloaded from the MODBASE database, which contains automatically generated homology models as part of the MODELER project [19]. Pathway information was obtained from KEGG PATHWAYS, MPMP (which is the primary malaria metabolic pathways site) [20] and Reactome [21]. Proteins were assigned pathway information by creating links to the pathways in the different databases they are involved in. The Enzyme Commission numbers were assigned to the proteins using enzyme data downloaded from the ENZYME database hosted by ExPASy [22]. For Plasmodium species, the EC numbers were obtained from PlasmoDB. The EC numbers were subsequently used to link proteins to the enzyme data in BRENDA [23] and KEGG ENZYME. Protein sequences from the DrugEBIlity database [24], which contains druggability predictions on protein structures from PDB, were kindly provided by the ChEMBL group. A sequence similarity search against the protein sequences from DrugEBIlity was carried out using NCBI BLAST, with an E-value cut-off of 10^-6^, in order to assess the druggability of the matching proteins in Discovery-2 as well as closely-related proteins. The hits were linked to the DrugEBIlity database for more detailed information on the druggability calculations. Data for protein-ligand interactions was collected from the ChEMBL database. The predictions were generated by performing a BLAST search against the ChEMBL protein targets and finding matching functional domains that were generated through the InterProScan program. Expression data is from the study performed by DeRisi *et al. *[25]. The literature mining was done on PubMed’s MEDLINE database abstracts (2012) by utilizing a form of the Aho-Corasick dictionary matching algorithm [26]. Articles are linked to proteins if they contain malaria-related keywords, and one of the protein’s aliases. Additionally, abstracts are scanned for relational keywords, such as "interacts with", to positively or negatively connect proteins with chemical compounds from ChEMBL. Uncommon English words that occur frequently in articles linked to proteins are also stored. An advanced search function was designed with the help of the Hibernate ORM (an object-relation mapper to provide a Java object interface to the relational database), together with a custom-built criteria library, to ensure the fastest possible filtering of protein sequences. Filtering can be done on various fields including function, orthology and literature. Chemical searches are performed using the MarvinSketch Applet, and JChemBase (ChemAxon).

## Results and discussion

### A brief introduction to the resource

#### Accession and search functions

Multiple approaches can be used to access the resource in retrieving the desired information. Data on a specific protein of interest may be obtained through a basic or an advanced search. In the basic search, a user may enter a PlasmoDB/Ensembl identifier, a UniProt accession number or alternatively perform the search using a protein name. A built in auto-complete feature in the protein identifier and UniProt accession searches assists users as they enter characters in the search space. The advanced search function allows the users to filter proteins using different search criteria in a step-by-step manner to select proteins matching their requirements. The search criteria for filtering protein sequences include organism, protein name, function, gene ontology, the availability of structural information, orthology, protein-ligand interactions, and the availability of related PubMed articles. A list of proteins matching the search criteria is returned from which the user may select the desired protein to view detailed information. The information for each protein is split across tabs representing different categories of annotation data. These tabs include “Summary”, “Function”, “Gene Ontology”, “Orthology”, “Structure”, “Metabolic Pathways”, “Interactions”, “Expression”, “Protein-Ligand Interactions”, “Druggability” and “Literature”. An additional tab, “Comments”, is available where users may submit comments related to a protein in the resource.

#### Summary

The “Summary” tab displays the summary information for a protein together with its amino acid sequence. The protein names and synonyms are displayed in this tab together with the protein identifier used in the source database as well as the UniProt accession and KEGG GENE identifier if available.

#### Function

The “Function” tab displays the predicted protein families, domains, and functional sites predicted using InterProScan. A graphical representation of the InterPro features found in the protein sequence and a table describing the InterProScan analysis are shown.

#### Gene ontology

The “Gene ontology” tab displays the GO terms assigned to the protein. The GO terms are categorized according to the three domains, i.e. cellular component, molecular function and biological processes.

#### Orthology

The results from the OrthoMCL clustering are displayed in the “Orthology” tab. In this tab the multiple sequence alignment of the protein sequences that form the cluster, generated with T-Coffee, is displayed in the JalView applet along with detail of the degree of conservation of the proteins.

#### Structure

The “Structure” tab displays the results from a BLAST search against the PDB database, as well as structural predictions from MODBASE.

#### Metabolic pathway

The “Metabolic Pathways” tab displays MPMP, KEGG and Reactome pathways that the protein is involved in, as well as enzyme information if available.

#### Interactions

The “Interactions” tab displays the predicted protein-protein interactions between the query protein and other proteins. The interactions are grouped as obtained from each of the three interaction databases (IntAct, MINT and DIP).

#### Protein-ligand interactions

The “Protein-ligand interactions” tab displays the result of BLAST searches and domain matches against the CHEMBL targets database, and allows inspection of the chemicals that interact with the CHEMBL blast hits.

#### Duggability

The “Druggability” tab displays the results of BLAST searches against domains and proteins sequences in the DrugEBIlity database.

#### Expression

The “Expression” tab displays the expression of the selected protein over the 48 hour intraerythrocytic developmental cycle (IDC).

#### Literature

The “Literature” tab shows PubMed abstracts related to the protein of interest.

#### Chemical searches function

Chemical searches may be performed by drawing a structure in the MarvinSketch applet, loading an existing structure or by providing a SMILES string. Substructure, superstructure, exact and similarity searches are available. Results are displayed in a tabular fashion and include the structure, ADMET properties and links to possible interacting proteins.

#### Clinical trials

An additional new feature is the “Clinical Trials” page which summarizes clinical trials related to malaria that are registered on the clinicaltrials.gov website, which is an NIH-maintained resource that provides easy access to information on publicly and privately supported clinical studies on a wide range of diseases and conditions. This feature in Discovery-2 allows a user to initiate searches into Discovery using current/experimental drugs as starting point. Compounds under investigation may be sent to the chemical search module to find exact or similar compounds, and possible interacting proteins may be selected.

### A sample investigation/Case study

To demonstrate the functionality available in Discovery-2, a sample investigation was performed on the enzyme S-adenosyl-L-homocysteine hydrolase (SAHH) from *P. falciparum.* PfSAHH is involved in cysteine and methionine metabolism, and has previously been proposed as a putative drug target [27]. The enzyme is responsible for the hydrolysis of S-adenosyl-L-homocysteine (SAH) to adenosine and L-homocysteine that is a reversible reduction/oxidation reaction involving nicotinamide adenine dinucleotide (NAD) as co-factor [27,28].

To search for PfSAHH in Discovery-2 using the basic search function, the protein entry could be retrieved by either using the PlasmoDB identifier PFE1050w, its UniProt accession P50250 or the enzyme name, S-adenosyl-L-homocysteine hydrolase. 

#### Summary

**Figure 1:**
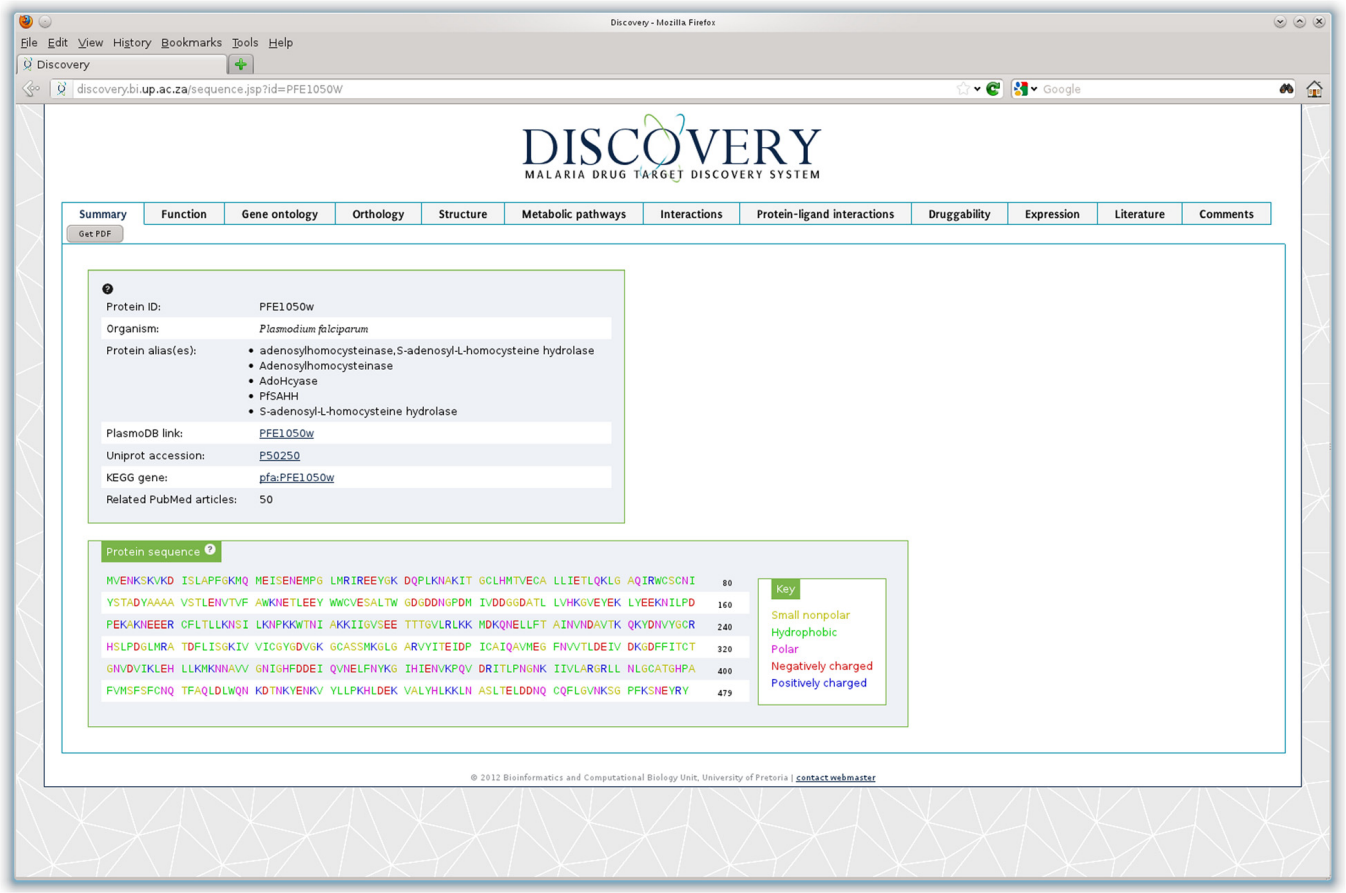
Summary view of the protein S-adenosyl-L-homocysteine hydrolase (PlasmoDB:PFE1050w), showing the basic protein information, links to other databases and protein sequence.

The "Summary" tab (Figure 1) provides the user with the protein identifier from PlasmoDB, VectorBase or Ensembl depending on the organism from which it originates. In addition known aliases for PfSAHH such as “adenosylhomocysteinase” and “AdoHcyase” are given. Links are provided to the PlasmoDB, Uniprot, the KEGG database pertaining to PfSAHH. Finally, the number of associated papers and the amino acid sequence are given.

#### Function

**Figure 2:**
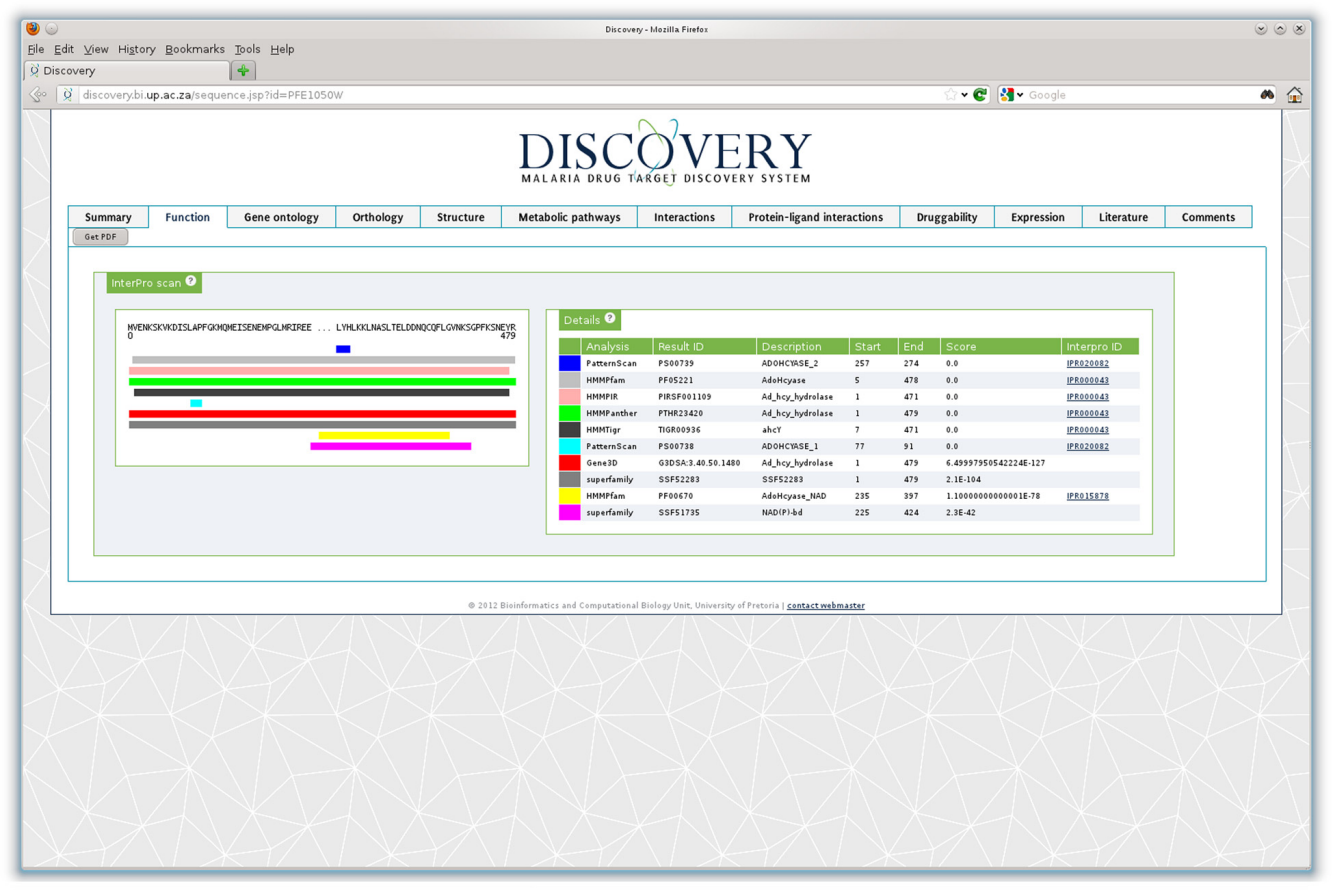
Functional prediction view of the protein S-adenosyl-L-homocysteine hydrolase (PlasmoDB:PFE1050w), showing a graphical overview as well as detail of domains and motifs found by InterProScan.

Results from the “Function” tab (Figure 2) showed three different InterPro identifiers that matched the query 1) InterPro:IPR020082 matched S-adenosyl-L-homocysteine hydrolase, conserved site, 2) InterPro:IPR015878 (S-adenosyl-L-homocysteine hydrolase, NAD binding domain and 3) InterPro:IPR000043 that matched Adenosylhomocysteinase. Table 1 summarizes the InterPro entries matching the *Pf*SAHH protein sequence.

**Table 1:** **Summary of the InterPro signatures matching to *Pf*SAHH**

**InterPro entry**	**Signatures**	**Analysis method**
IPR000043 (Family)	TIGR00936 (ahcY)	HMMTigr
	PF05221 (AdoHcyase)	HMMPfam
	PTHR23420 (Ad_hcy_hydrolase)	HMMPanther
	PIRSF001109 (Ad_hcy_hydrolase)	HMMPIR
IPR015878 (Domain)	PF00670 (AdoHcyase_NAD)	HMMPfam
IPR020082 (Site)	PS00738 (ADOHCYASE_1)	PatternScan
	PS00739 (ADOHCYASE_2)	PatternScan

#### Gene ontology

Two GO terms, GO:0004013 (adenosylhomocysteinase activity) and GO:0016787 (hydrolase activity) were found to be associated with PfSAHH at the molecular function level whereas GO:0006730 (one-carbon metabolic process) is associated with the protein at a biological process level. No GO terms were found to be associated with PfSAHH at cellular component level.

#### Orthology

**Figure 3:**
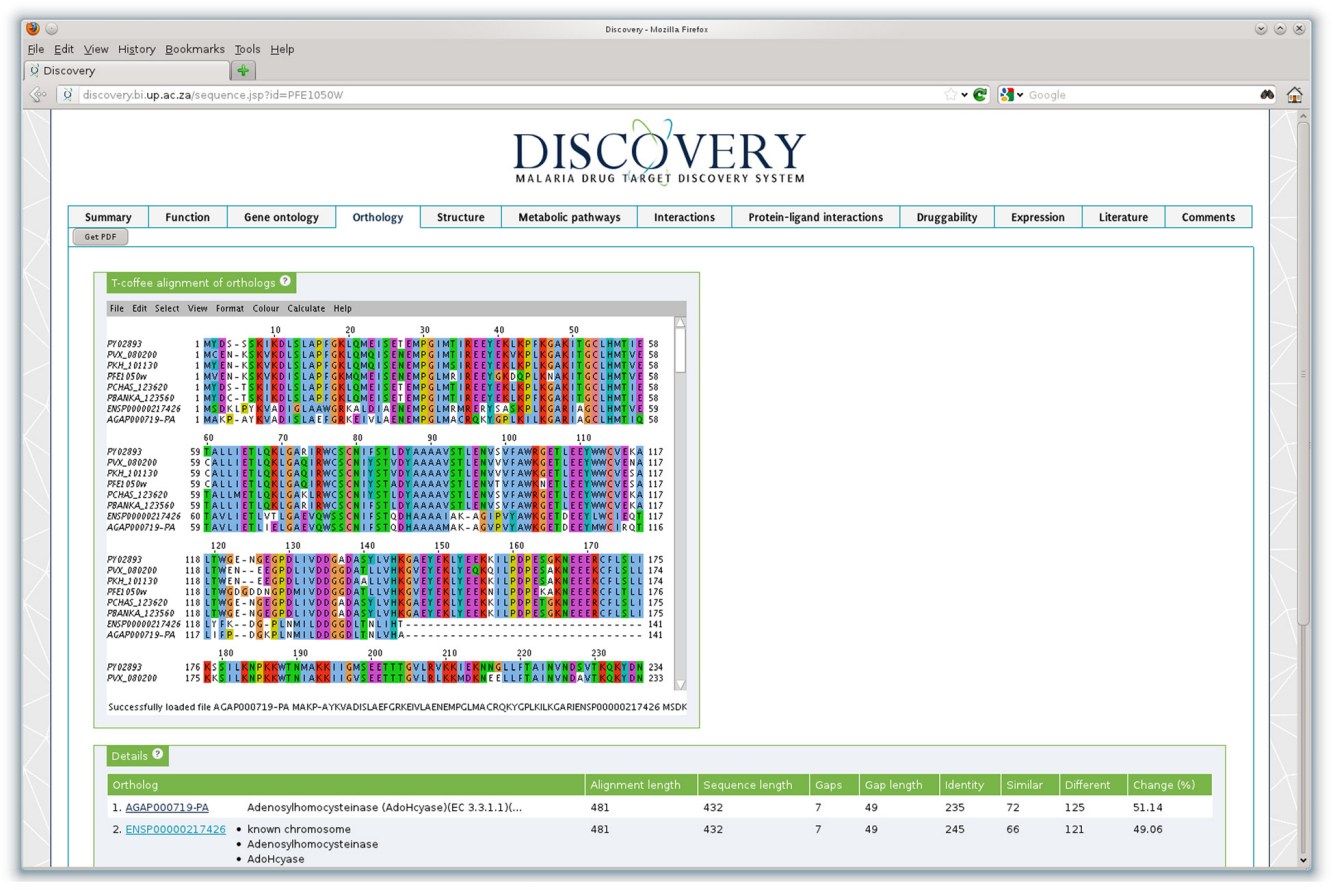
Orthology information for S-adenosyl-L-homocysteine hydrolase (PlasmoDB:PFE1050w), showing a T-Coffee multiple alignment of the orthologs predicted for SPfSAHH in the other malaria species as well as the human and mosquito hosts.

OrthoMCL clustering (Figure 3) showed SAHH to be present in all eight species studied within Discovery 2. From the Ortholog table it was noted that *A. gambiae*, and *H. sapiens* had the same alignment length to the Plasmodium species but differed in their sequence length. *A. gambiae*, and *H. sapiens* showed 7 gaps with a total gap length of 49 amino acids. From the T-coffee alignment an insert of 42 residues were identified in all the Plasmodium species starting at residue 145 in *P. falciparum*.

#### Structure

A BLAST search of the PfSAHH protein sequence against the PDB identified an exact protein sequence match for PfSAHH (PDB:1V8B). Related protein structures that have 70% coverage with the PfSAHH sequence are provided together with an alignment between the target and template. In addition two MODBASE models have been constructed for the PfSAHH protein, PFE1050w.1 and PFE1050w.2 which were built on templates PDB:1LI4 and PDB:1V8B, respectively.

#### Metabolic pathways

The “Metabolic Pathways” tab showed PfSAHH as adenosylhomocysteinase with its EC number, 3.3.1.1. The MPMP database showed the PfSAHH enzyme to be associated with methionine and polyamine metabolism, proteins targeted by the thioredoxin superfamily enzymes and S-glutathionylated proteins. The KEGG pathway associated the enzyme with cysteine and methionine metabolism and found it to be involved in various metabolic pathways (non-specific global map of all metabolic pathways) which include glycan biosynthesis, lipid, carbohydrate, amino acid, nucleotide, co-factor, vitamin, terpenoids, polyketide and energy metabolism. Reactome showed PfSAHH to be involved in metabolism of amino acids and derivatives as well as biological oxidations. The metabolic pathway function provides the user with an overview of the metabolic relevance of the target of interest.

#### Protein-protein interactions

The "Interactions" tab reports protein-protein interactions and had interaction data associated with PfSAHH within the IntAct and MINT databases. Nine interactions were associated with PfSAHH including itself. The UniProt accessions for these proteins that interact with the PfSAHH enzyme are UniProt:Q8I561 (conserved protein, unknown function), UniProt:Q8I2F7 (ring-exported protein [REX3]), UniProt:Q8IFP1 (U5 small nuclear ribonucleoprotein-specific protein, putative), UniProt:O96221 (Sec31p putative), UniProt:Q8IKB6 (histone deacetylase, putative), UniProt:Q8IIC8 (conserved protein, unknown function), UniProt:Q8IBL5 (conserved protein, unknown function), UniProt:Q8IAZ3 (Eukaryotic translation initiation factor 3 subunit G) and UniProt:Q8IJY1 (conserved protein, unknown function). All identified interactions were determined by the use of a two hybrid system that uses transcriptional activity as a measure of protein-protein interactions with all interactions therefore being demonstrated by physical associations.

#### Protein-ligand interactions

**Figure 4:**
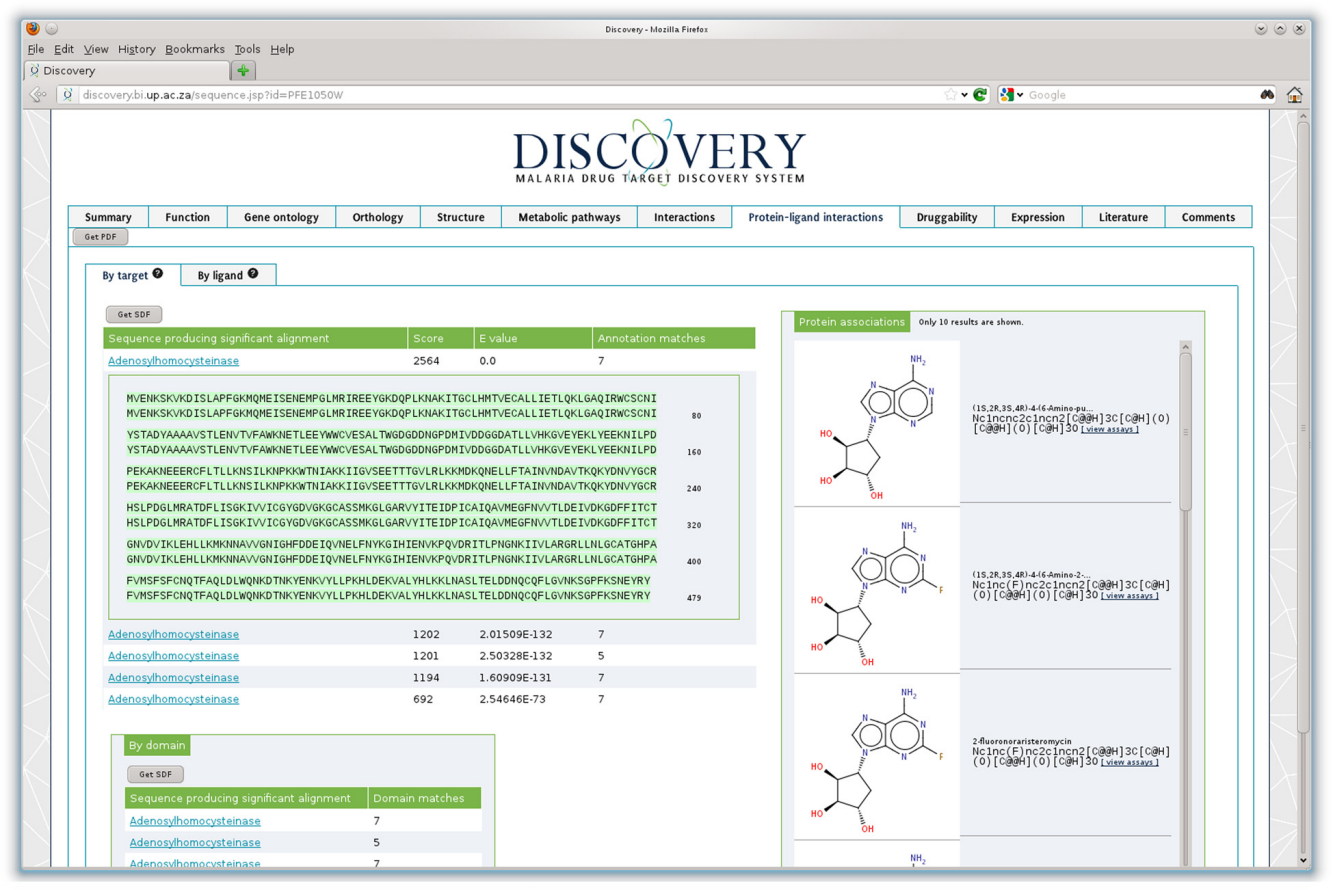
**Protein-ligand interaction view for S-adenosyl-L-homocysteine hydrolase (PlasmoDB:PFE1050w), showing predicted interactions with a series of ligands, based on a BLAST hit to ChEMBL.** An alignment to the BLAST hit is also shown.

Protein-ligand interactions were identified by a BLAST search against the CHEMBL targets database, and were reported in the targets section with the ligands associated in the ligand section (Figure 4). PfSAHH was identified from the CHEMBL targets database along with SAHH from other organisms including *Mus musculus*, *Homo sapiens* and *Thermotoga maritima,* all showing good BLAST hits. Domain matching of PfSAHH showed no additional matches compared to the BLAST results for the complete protein sequence. Two hundred and ninety-one chemical compounds were identified which have been tested for activity against PfSAHH and its homologs, 819 respective bioactivities involving those compounds were found.

#### Druggability

Druggability data for PfSAHH, showed that the molecules with the most significant BLAST domain matches from DrugEBIlity (PfSAHH crystal structure and PDB:1V8B) are not predicted to be druggable, although tractability is indicated. From the BLAST alignments PfSAHH (UniProt:P50250) had 0% overall druggability whilst the top two homologues UniProt:P60176 (SAHH from Mycobacterium tuberculosis) and UniProt:Q3JY79 (SAHH from *Burkholderia pseudomallei*, strain 1710b) had druggability scores of 48% and 45%, respectively.

#### Expression

The results in the expression view indicate that the protein is expressed predominantly in the trophozoite stage and is under-expressed in the early ring stage, indicating that it may have a role in parasite growth and preparation for division.

#### Literature

The literature functionality showed 26 articles related to SAHH from PubMed abstracts. The compounds cysteine, adenosine, aristeromycin, and ilimaquinone are shown as the main possible interactors with SAHH based on text relation mining of abstracts.

In summary, it was possible to demonstrate by means of a single search that the protein PfSAHH has ahcY, AdiHcyase and Ad_hcy_hydrolase signatures and that orthologs are found in the other Plasmodium species as well as the human and mosquito hosts. The Plasmodium enzymes have insertions in relation to the hosts’ enzymes, and a crystal structure as well as homology models are available for the *P. falciparum* enzyme. Experimental protein-protein interaction evidence is available, and despite a relatively weak druggability score, a wide range of compounds that potentially interact with the enzyme are highlighted.

### A sample investigation using a chemical structure

As a brief example of a compound-based search using a known anti-malarial drug, the SMILES string for pyrimethamine was provided, and an exact search was performed. The compound 5-(4-chlorophenyl)-6-ethylpyrimidine-2,4-diamine (pyrimethamine) was retrieved as the only hit. The compound information view provided detail about the molecular mass, hydrogen bond donor count, acceptor count, logP, logD, ring count and rotatable bond count, which are all relevant properties for the selection of druggable compounds. The compound passed the Lipinski rule of five [29]. When a search for putative targets was performed, a series of dihydrofolate reductase and bifunctional dihydrofolate reductase – thymidylate synthase enzymes were found in all the relevant species. This included the *Plasmodium falciparum* drug target, PFD0830w.

## Conclusions

Discovery-2 is an update of the original Discovery resource, and is a complete rewrite of the system utilizing an updated database structure and new software technologies, and underlying functionality to perform automated updates using web services. A series of new features have been added such as relations to targets and compounds in ChEMBL, links to druggability information, automated literature links and relations to compounds in clinical trials.

The resource allows researchers to perform quick searches based on specific proteins, but to also perform mining and filtering of possible drug targets based on a series of molecular properties. Results provide the researcher with information about the protein’s functional annotations, ontology terms, orthology to the different malaria parasite species and host proteins, structural information, pathway information, protein and ligand interaction information, druggability information and literature links. These result categories were selected to optimally assist researchers in selecting proteins for further detailed *in silico* and experimental investigation to assess the suitability of the protein as a putative drug target.

It is hoped that this resource enables the malaria community to quickly and easily obtain information related to the suitability of a protein as a drug target, and to perform the *in silico* selection of proteins from the malaria genome to effectively reduce the data space for further detailed experimentation.

### Availability

Users can access the Discovery-2 resource at http://discovery.bi.up.ac.za.

## Competing interests

The authors declare that they have no competing interests.

## Authors’ contributions

FJ is the PI and grant holder. Core functionality was developed by JS. The protein-related functionality was developed by JS and PM. The chemical-related functionality was developed by MS and JS. Web-services and scoring was developed by JS, MLG and PB. All authors contributed to the writing of the manuscript. All authors have read and approved the final manuscript.
